# Comparative Efficacy of Interleukin-7 and -15 Blockade in Alleviating Experimental Chronic Uveitis and Suppressing Pathogenic Memory CD4^+^ T Cells

**DOI:** 10.1167/iovs.66.5.9

**Published:** 2025-05-05

**Authors:** Qiurong Zhu, Maryam Shayan, Rachel M. Huckfeldt, Yihe Chen

**Affiliations:** 1 Schepens Eye Research Institute of Massachusetts Eye and Ear, Department of Ophthalmology, Harvard Medical School, Boston, Massachusetts, United States; 2 Massachusetts Eye and Ear, Department of Ophthalmology, Harvard Medical School, Boston, Massachusetts, United States

**Keywords:** chronic uveitis, immunological memory, CD4^+^ T cells, IL-7, IL-15

## Abstract

**Purpose:**

We have previously demonstrated the pathogenic function of memory CD4^+^ T cells, which express IL-7 receptor (IL-7R) and IL-15R, in experimental chronic autoimmune uveitis (CAU). Here, we aimed to compare the therapeutic efficacy of blocking IL-7 or IL-15 in CAU.

**Methods:**

C57BL/6J mice were induced for CAU, then intraperitoneally injected with an anti-IL-7 antibody (Ab), an anti-IL-15 Ab, or an IgG control for 2 weeks. Disease was evaluated by weekly fundoscopy, optical coherence tomography (OCT), and full-field electroretinography for four weeks from the initiation of treatment. At week 4, retina and cervical lymph nodes (CLN) were collected for flow cytometry analysis of T-cell response.

**Results:**

The anti-IL-7 Ab led to progressively reduced retinal infiltration and structural damage, with rapid recovery of retinal function. The anti-IL-15 Ab resulted in moderately reduced retinal infiltration and structural damage, along with a delayed, partial functional improvement. Compared to the control group, the anti-IL-7 Ab group exhibited significantly reduced disease scores from baseline on fundoscopy and OCT at week 4, and substantially improved dark-adapted (DA) a-wave and light-adapted b-wave responses at week 2; although the anti-IL-15 Ab group showed significantly improved disease from baseline only on OCT and increased DA b-waves at week 4. Both treatments effectively depleted the retinal infiltrating T cells and reduced memory Th17 cells in the CLN.

**Conclusions:**

Our proof-of-concept study demonstrates that blocking IL-7 or IL-15 leads to specific depletion of the uveitogenic memory CD4^+^ T cells and disruption of disease chronicity in uveitis.

Noninfectious uveitis is characterized by immune-mediated intraocular inflammation that is often treatment-resistant and presents a chronic relapsing course, leading to significant vision impairment and poor quality of life.^1^^–^^4^ It is estimated that uveitis causes about 30,000 new cases of legal blindness per year in the United States and accounts for approximately 10% of irreversible vision loss.^5^^–^^8^ Currently, corticosteroids remain the first-line mainstay of treatment for this blinding condition but bear inherent serious side effects, including local (raised intraocular pressure and cataract) and systemic (such as increased blood pressure and glucose) morbidities that limit their prolonged use for treating chronic uveitis.^9^^,^^10^ Nonsteroidal, broad immunosuppressants have been used to control inflammation or reduce the usage of corticosteroids; however, their use is also associated with various common toxic effects and needs constant monitoring during the therapy.^11^^,^^12^ Critically, these treatments are nonspecific to the disease, and they cannot cure the disease but only limit the intraocular inflammation, and thus they often cannot effectively prevent disease recurrence.

The advent of the classic experimental autoimmune uveitis (EAU) model in mice over 30 years ago^13^ has greatly improved our understanding of the immunopathogenesis of noninfectious uveitis and facilitated the successful translation of preclinical therapeutics to human use.^14^ Investigation using this model has ultimately led to the approval by the Food and Drug Administration of tumor necrosis factor-α (TNF-α) inhibitor as the first targeted, corticosteroid-sparing biologic for treating noninfectious uveitis involving the intermediate or posterior eye.^15^^–^^17^ Despite being approved, the TNF-α inhibitor in clinical trials still failed to achieve the primary efficacy endpoint (prolonged time to treatment failure) in more than 50% of treated patients,^17^ suggesting that TNF-α is likely not the most critical mediator underlying chronic uveitis. In addition, anti-TNF-α treatment caused serious side effects, including cancers and infections,^17^^–^^19^ indicating that universal suppression of TNF-α may compromise the normal function of the immune system. Recent efforts in developing more specific biologic agents for chronic uveitis have been focused on IL-17, encouraged by the promising pre-clinical data from EAU studies demonstrating the dominant role of T helper-17 (Th17) cells, the primary IL-17 producer, in the pathogenesis of EAU, and the effectiveness of neutralizing IL-17 in ameliorating disease severity of EAU.^20^^,^^21^ However, several independent phase 3 clinical trials evaluating subcutaneous injection of an anti-IL-17 antibody (Secukinumab) for chronic noninfectious uveitis involving the intermediate or posterior segment of the eye failed to meet the primary efficacy endpoint which was uveitis recurrence during withdrawal of concomitant standard nonspecific immunosuppressive medication.^22^ Later, a smaller phase 2 clinical trial showed that intravenous delivery of Secukinumab led to higher drug concentrations and a better response rate, but it was still poor in achieving sustained disease remission.^23^ In addition to IL-17, the other Th17 pathway cytokine IL-23 has been intensively investigated, given its critical role in activating Th17 cells,^24^ up-regulation of IL-23 in human uveitis,^25^ and well-demonstrated therapeutic effects of inhibiting IL-23 in EAU.^26^ To date, several small-scale, nonrandomized clinical trials assessing an anti-IL-23 antibody (Ustekinumab) in chronic uveitis have been completed, and yet no publications are available showing therapeutic effects of Ustekinumab in chronic uveitis.^27^ These setbacks in translating Th17 pathway in non-infectious uveitis have made us revisit those studies performed in EAU models: they have primarily focused on the acute peak of disease after induction (two to three weeks), with few having investigated the chronic phase of the disease, which is, however, the major focus in human research.

To better understand the cellular and molecular immunopathogenesis of human chronic uveitis, we have developed a robust chronic autoimmune uveitis (CAU) in mice, which exhibits chronic chorioretinitis for over three months with disruption of the outer blood-retina barrier, along with impaired retinal structures and function.^28^ Interestingly, mice with CAU show persistent infiltration of CD4^+^ T cells in the retina, and these T cells present with a unique “memory phenotype” characterized by high expression of receptors for IL-7 and IL-15 (IL-7R and IL-15R). Importantly, these memory CD4^+^ T cells demonstrate prolonged survivability and critical uveitogenicity, supporting their important functions in sustaining a protracted course of intraocular inflammation in uveitis.^29^ In the present study, we have aimed to specifically deplete these pathogenic memory CD4^+^ T cells in mice with CAU by neutralizing IL-7 or IL-15 that are functionally critical to the maintenance of memory T cells,^30^^,^^31^ to evaluate the therapeutic potential of this novel strategy in disrupting the chronicity of uveitis.

## Methods

### Animals

Wild-type C57BL/6J mice (Strain no. 000664; The Jackson Laboratory, Bar Harbor, ME, USA) at eight to 10 weeks of age were used for this study. All animal experiments were approved by the Schepens Eye Research Institute Animal Care and Use Committee and adhered to the Association for Research in Vision and Ophthalmology Statement for the Use of Animals in Ophthalmic and Vision Research.

### Experimental CAU

Chronic uveitis was induced in mice using our established CAU protocol.^28^ In brief, mice were immunized with 150 µg human interphotoreceptor retinoid-binding protein peptide (residues 161-180, Cat no. AS-60183; AnaSpec, Fremont, CA, USA) and 300 µg human interphotoreceptor retinoid-binding protein peptide (residues 1–20, Cat no. AS-62297; AnaSpec) emulsified in 0.2 mL CFA (containing 2.5 mg/mL Mycobacterium tuberculosis strain H37RA, Difco) (Cat no. F5881; Sigma-Aldrich Corp., St. Louis, MO, USA), along with injection of 0.2 µg Bordetella pertussis toxin (Cat no. P7208; Sigma-Aldrich Corp.). Animals at 12 weeks or later after immunization were used as the chronic model. The immunization was performed via subcutaneous injection of both flanks (50 µL each side), as well as the base of the tail (100 µL).

### Digital Fundus Imaging and Scoring

A Micron IV (Phoenix, Pleasanton, CA, USA) retinal imaging system was used to take fundus photographs. Mice were anesthetized using ketamine (NDC no. 17033-100-10; Dechra Veterinary Products, Northwich, UK) and xylazine (NDC no. 59399-110-20; Akorn Pharmaceuticals, Gurnee, IL, USA) (100 mg/kg + 20 mg/kg, respectively), and pupils were dilated using 0.5% tropicamide (NDC no. 24208-585-64; Bausch & Lomb, Rochester, NY, USA). Eyes were kept moist by application of ocular lubricant (Genteal gel; Alcon, Fort Worth, TX, USA), and were examined for optic disc inflammation, retinal vessel changes, retinal infiltrates, and structural damage. Fundus images were scored by a masked investigator using a grading system (on a scale of 0-4) that provides detailed and refined differentiation on the severity of chronic disease, which primarily presents with diffuse retinal infiltrates (Table 1), modified from previous studies that mainly focused on the acute uveitis.^28^^,^^32^^,^^33^

**Table 1.** Scoring Scale for Evaluating Chronic Uveitis on Fundus Images

Score	Retinal Tissue Infiltrates
0	No lesions
1	1–15 small lesions
2	16–30 small lesions
3	31–50 small lesions
4	>50 small lesions or linear/confluent lesions

### Retinal Imaging by Spectral Domain Optical Coherence Tomography

After anesthesia and pupil dilation as detailed above, mice were restrained in a mounting tube fixed on a six-axis platform. Genteal gel was applied to both eyes to prevent drying of the cornea. A spectral domain optical coherence tomography (OCT) system (Bioptigen, Durham, NC, USA) was used for in vivo noncontact imaging of eyes. B scan was obtained with images centered on the optic nerve head. The hyperreflective foci (HRF) were defined as discrete and well-circumscribed dots or roundish lesions (no smaller than 10 µm to exclude noise signals) within retinal layers with reflectivity comparable to the retinal nerve fiber layer or retinal pigment epithelium (RPE) layer,^34^^,^^35^ and counted on 360 continuous frames of B-scan images centered on optic nerve head within a 600 µm radius of area; and larger lesions of more than 20 µm were referred to as hyperreflective clumps. OCT images were graded on a scale of 0-4 (Table 2) modified from previous studies by a masked grader.^33^^,^^36^^,^^37^

**Table 2.** Scoring Scale for Evaluating Chronic Uveitis on OCT Images

Score	Retinal HRF and Structural Damage
0	<5 small (10-20 µm) HRF
1	5–10 dot shaped or roundish HRF
2	11–20 dot shaped or roundish HRF
3	20–30 dot shaped or roundish HRF or 1 large (>20 µm) clump
4	>30 dot or roundish HRF or >1 large clump or disruption of retinal lamination

### Electroretinography

After overnight dark adaptation, mice were prepared for electroretinography (ERG) recording under dim red light. After anesthesia and pupil dilation as detailed above, one drop of 0.9% sterile saline was applied to the cornea to prevent dehydration and to allow electrical contact with the recording electrode (gold wire loop). A 25-gauge platinum needle, inserted subcutaneously in the forehead, served as the reference electrode, while a needle inserted subcutaneously near the tail served as the ground electrode. A series of flash intensities were produced by Espion Ganzfeld (Diagnosys, Lowell, MA, USA) to test both scotopic (dark-adapted) and photopic (light-adapted) responses. The major ERG components (a-wave and b-wave) were measured using the Espion software (Diagnosys).^36^^,^^38^ The a-wave amplitude was measured from the baseline to the trough of the a-wave, and the b-wave amplitude was measured from the trough of the a-wave to the peak of the b-wave.

### In Vivo Treatment of Animals With Antibodies (Ab)

Mice with established CAU were intraperitoneally injected with 100 µl an anti-IL-7 Ab (100 µg, Cat #: AB-407-NA, R&D Systems, Minneapolis, MN, USA),^39^ an anti-IL-15 Ab (50 µg, Clone no. AIO.3, Cat no. 16-7154-85; eBioscience, San Diego, CA, USA),^40^^,^^41^ or goat IgG control (100 µg, Cat no. AB-108-C; R&D Systems) every other day for two weeks for total eight injections.

### Flow Cytometry Analysis and Reagents

Before tissue collection, mice were deeply anesthetized and underwent transcardial perfusion with PBS to remove circulating immune cells. Briefly, a small incision was made in the chest to access the heart, and a needle was inserted into the left ventricle to deliver 20 to 30 mL of heparinized (10 IU/mL) PBS at a controlled, steady rate to avoid pressure-related damage. Simultaneously, an opening was created in the right atrium to allow drainage of blood and perfusate. Perfusion was continued until the liver and lungs became visibly pale, confirming the clearance of blood, after which the infusion was stopped, and death was verified. After enucleation, retinal tissues were carefully isolated and subjected to enzymatic digestion using 1 mg/mL Collagenase D. The digestion process was conducted at 37°C for 45 minutes to facilitate the dissociation of retinal cells while maintaining cellular viability for flow cytometry. After digestion, the cell suspension was passed through a 70-µm strainer to remove tissue debris and obtain a single-cell suspension optimized for flow cytometric analysis. In addition, eye-draining cervical lymph nodes (CLN, both superficial and deep) and spleens of mice were collected, and single-cell suspensions were prepared using a 70-µm cell strainer (BD Biosciences, Franklin Lakes, NJ, USA). The following dye (Invitrogen, Carlsbad, CA, USA) and antibodies (BioLegend, San Diego, CA, USA) were used for flow cytometry analysis: Fixable Viability Dye eFluor 660 (Cat no. 65-0864; Invitrogen), Brilliant Violet 421-conjugated anti-CD4 (clone RM4-5, Cat no. 100544), FITC-conjugated anti-CD3 (clone 145-2C11, Cat no. 100305), PerCP/Cy5.5-conjugated anti-CD44 (clone IM7, Cat no. 103032), and APC-conjugated anti-IL-17 (clone TC11-18H10.1, Cat no. 506916). For intracellular IL-17 staining, cells were stimulated with 50 ng/mL phorbol 12-myristate 13-acetate and 500 ng/mL ionomycin (Cat no. P8139 and I0634; Sigma-Aldrich) for six hours at 37°C and 5% CO_2_ in the presence of GolgiStop (4 µL per 6 mL cell culture, Cat no. 554724; BD Biosciences) to inhibit cytokine secretion. Stained cells were examined with an LSR II flow cytometer (BD Biosciences), and the results were analyzed using FlowJo software (version 10.1; Tree Star, Ashland, OR, USA).

### Statistical Analyses

One-way ANOVA was used to compare three groups, and *t*-tests were further used to perform the pairwise comparisons. The *P* values were corrected using the Benjamini-Hochberg false discovery rate for multiple comparisons. Mixed-effects models were used to evaluate the change over time. Week 0 was taken as the reference group, and weeks 1 to 4 were compared with the reference group. Animal IDs were used as random effects to adjust for within-individual and between-individual variance. Dunnett's tests were used to correct *P* values for multiple time points. Data are summarized as mean ± SEM. All statistical analyses were performed with Prism software (version 10; GraphPad Software, San Diego, CA, USA), and differences were considered significant at *P* < 0.05.

## Results

### Neutralization of IL-7 Is More Effective Than Neutralization of IL-15 in Reducing Retinal Infiltration on Fundoscopy

**Figure 1.**
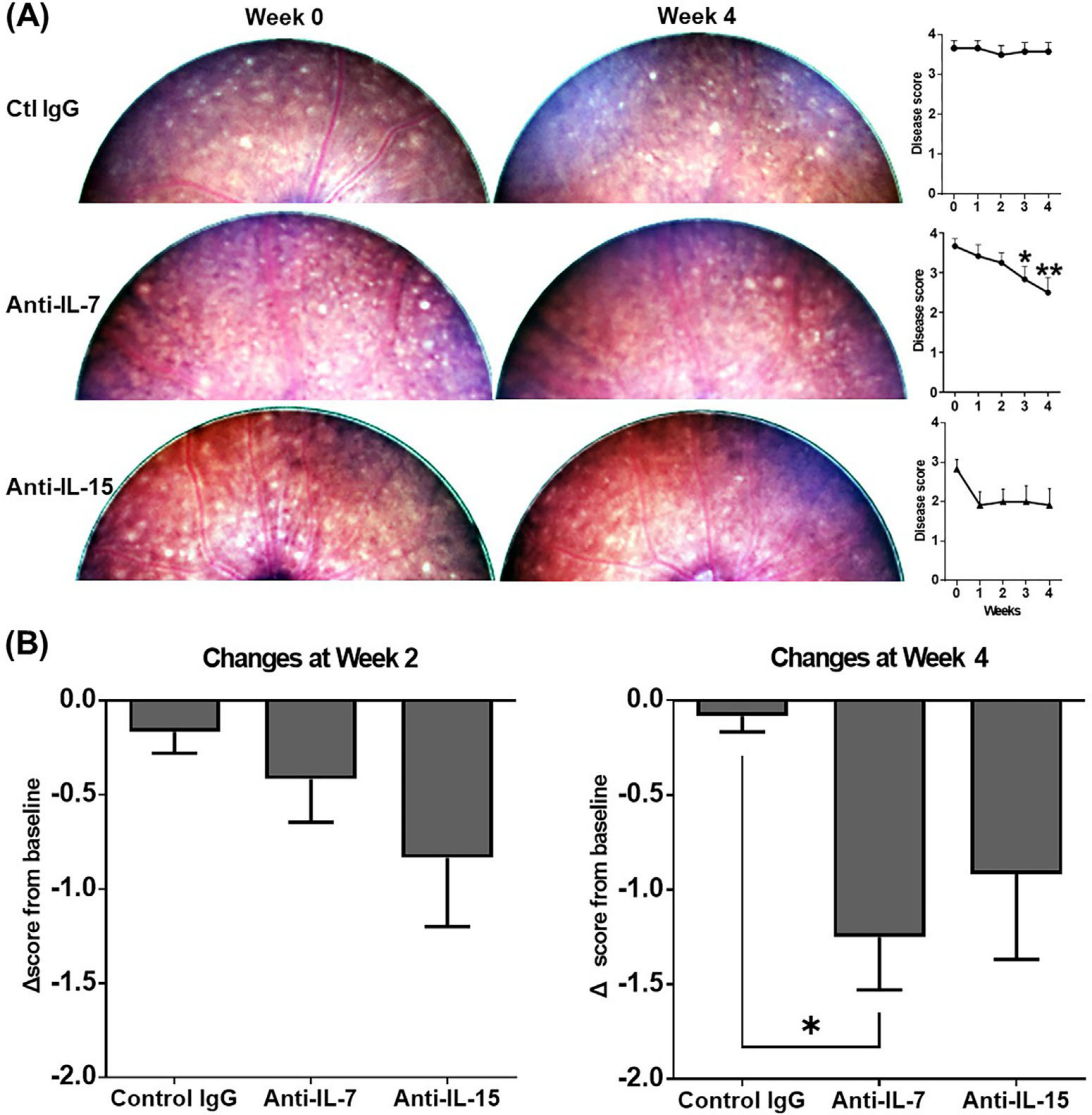
Fundoscopic evaluation of chronic uveitis disease activity. **(A)** Mice with CAU were treated with control IgG, anti-IL-7 Ab, or anti-IL-15 Ab from week 0–2, and digital fundus images were taken every week from week 0–4. Representative digital fundus images at baseline (week 0) and week 4 from the same animals in each group are shown on the left, and the scores (on a scale of 0–4) are summarized in the bar graphs on the right. **(B)** Changes in disease scores at weeks 2 and 4 from the baseline are summarized in the bar graphs. Data shown are mean ± SEM of pooled three independent experiments. n = 12/group; *P < 0.05, **P < 0.01 compared to week 0 **(A)** or indicated groups **(B)**.

Mice with CAU showed multiple small retinal lesions (a few of them formed perivascular cuffing) on fundoscopic observations, with average disease scores of 3-4 (on a scale of 0-4) among groups. Following the 2-week treatment, the anti-IL-7 Ab group showed progressive disease reduction and the disease scores were significantly lower than the baseline at week 4 with an average score of 2; the anti-IL-15 Ab group showed a trend of disease reduction but the disease scores were not significantly different from the baseline; in contrast, the disease scores of control IgG group remained the same level as the treatment baseline (Fig. 1A). At the end of the treatment (week 2), neither anti-IL-7 nor anti-IL-15 Ab treatment led to significant disease reduction compared to the control IgG treatment; however, two weeks after the end of the treatment (week 4), the anti-IL-7 Ab treatment showed significant disease reduction from the baseline than the control IgG group (means of Δ scores: −1.3 vs. −0.1) whereas the anti-IL-15 Ab treatment only led to a moderate disease reduction (means of Δ scores: −0.9) (Fig. 1B).

### Neutralization of IL-7 or IL-15 Leads to Comparably Sustained Improvement of Damaged Retinal Structures on OCT

**Figure 2.**
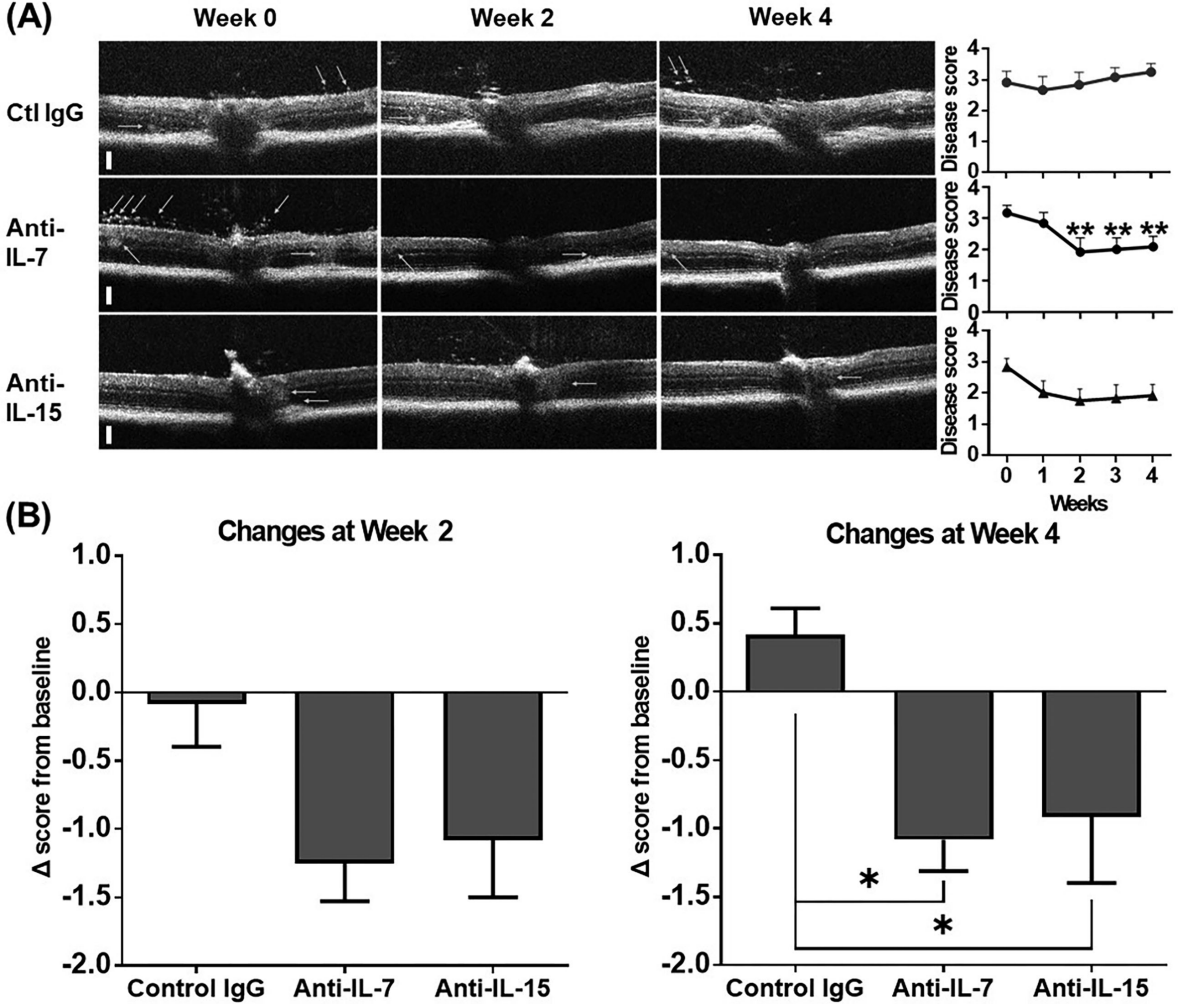
OCT assessment of retinal structures in chronic uveitis. **(A)** Mice with CAU were treated with control IgG, anti-IL-7 Ab, or anti-IL-15 Ab from week 0–2, and OCT B-scan images were taken every week from week 0–4. Representative images at baseline (week 0) and week 4 from the same animals in each group are shown on the left, and the scores (on a scale of 0–4) are summarized in the bar graphs on the right. Arrows indicate hyperreflective vitreous opacities or large retinal hyperreflective clumps along with disrupted lamination. *Vertical scale bars* = 100 µm. **(B)** Changes in disease scores at weeks 2 and 4 from the baseline are summarized in the bar graphs. Data shown are mean ± SEM of pooled three independent experiments. *n* = 12/group; *P < 0.05, **P < 0.01 compared to week 0 **(A)** or indicated groups **(B)**.

Mice with CAU showed relatively mild vitreous infiltration but significant retinal structural damages including multiple HRF, an indicator of inflammation,^29^^,^^34^ and disruption of retinal lamination on OCT examination.^28^ The HRF were discrete and well-circumscribed dots or roundish lesions (10-20 µm) within retinal layers with reflectivity comparable to the retinal nerve fiber layer; when the hyperreflective lesions were larger (>20 µm) confluent clumps, they were preferentially located in the outer retina with reflectivity similar to the RPE layer but also observed in the inner nuclear layer and inner plexiform layer. After treatment, the anti-IL-7 Ab group showed significantly diminished HRF and improved laminated structures on the B-scans as early as week 2, and this effect persisted even after the treatment stopped until the end of the follow-up (week 4). The anti-IL-15 Ab treatment led to a fast and moderate improvement by week 1, which was sustained by week 4. In contrast, the control IgG treatment did not result in any improvement in HRF or retinal structures (Fig. 2A). At the end of the treatment (week 2), both the anti-IL-7 Ab and anti-IL-15 Ab treatment led to disease reduction compared to the control IgG treatment, and the effect persisted for another 2 weeks after the termination of treatment (i.e., week 4 at the end of our follow-up) and reached statistical significance (means of Δ scores at week 4 for the anti-IL-7 Ab, anti-IL-15 Ab, and control IgG: −1.1, −0.9, vs. 0.4) (Fig. 2B).

### Neutralization of IL-7 Improves the Impaired Retinal Function More Rapidly Than Neutralization of IL-15

**Figure 3.**
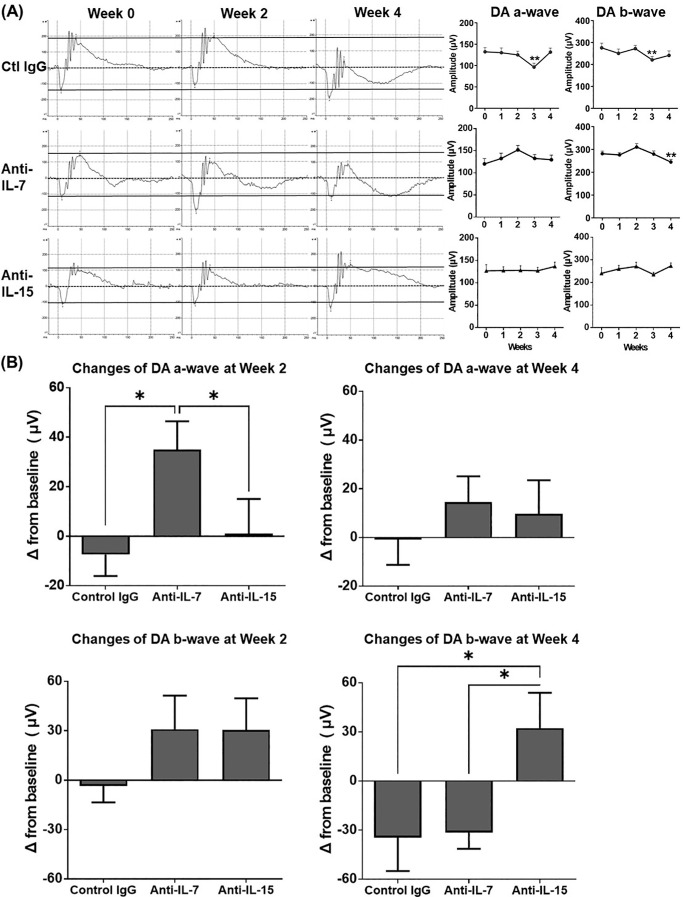
ERG evaluation of DA retinal responses in chronic uveitis. **(A)** Mice with CAU were treated with control IgG, anti-IL-7 Ab, or anti-IL-15 Ab from week 0–2, and ERG responses from DA eyes were recorded every week from week 0–4. Representative ERG responses to light stimuli at 24.1 cd s/m^2^ from the same animals in each group are shown on the left. The a-wave amplitude was measured from the baseline to the trough of the a-wave, and the b-wave amplitude was measured from the trough of the a-wave to the peak of the b-wave. *Straight solid lines* across the weeks serve as references indicating the baseline (week 0) measurement of a-wave and b-wave. Amplitudes are depicted in bar charts on the right. **(B)** Changes in amplitudes at weeks 2 and 4 from the baseline are summarized in the bar graphs. Data shown are mean ± SEM of pooled three independent experiments. *n* = 12/group; *P < 0.05, **P < 0.01 compared to week 0 **(A)** or indicated groups **(B)**.

**Figure 4.**
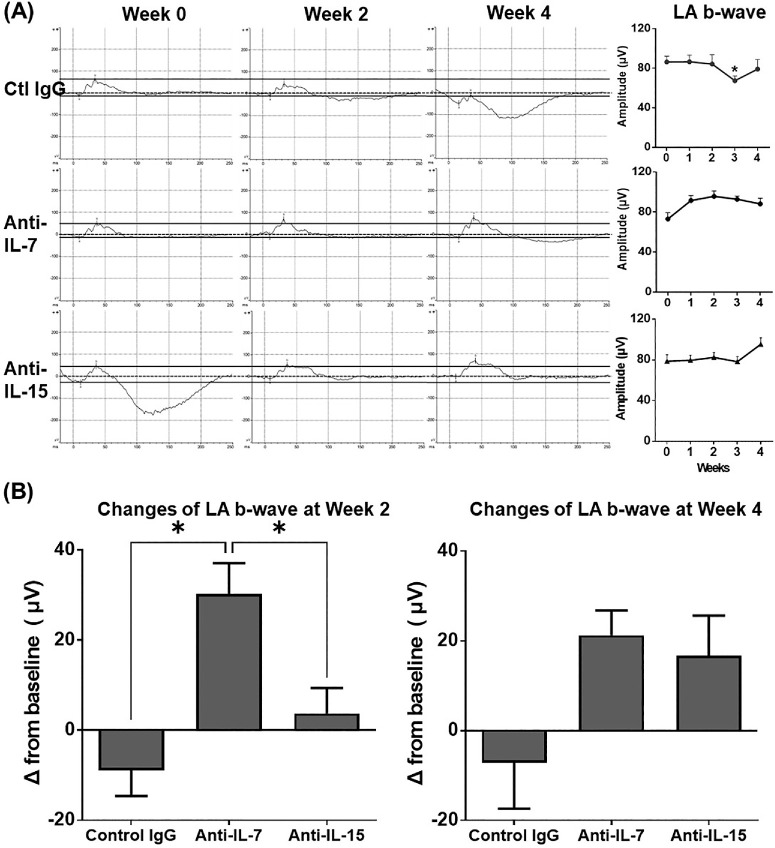
ERG evaluation of light-adapted (LA) retinal responses in chronic uveitis. **(A)** Mice with CAU were treated with control IgG, anti-IL-7 Ab, or anti-IL-15 Ab from week 0–2, and ERG responses from LA eyes were recorded every week from week 0–4. Representative ERG responses to light stimuli at 25.6 cd s/m^2^ from the same animals in each group are shown on the left. *Straight solid lines* across the weeks serve as references indicating the baseline (week 0) measurement of a-wave and b-wave. Amplitudes are depicted in bar charts on the right. **(B)** Changes in amplitudes at weeks 2 and 4 from the baseline are summarized in the bar graphs. Data shown are mean ± SEM of pooled three independent experiments. *n* = 12/group; *P < 0.05 compared to week 0 **(A)** or indicated groups **(B)**.

Mice with CAU are characterized by impaired retinal function evidenced by significantly decreased amplitudes of dark-adapted (DA) a-waves, DA b-waves, and light-adapted (LA) b-waves on the full-field ERG.^28^ After treatment, the anti-IL-7 Ab group showed moderately increased amplitudes of DA a-waves and b-waves and LA b-waves by week 2, followed by a decline toward baseline level by week 4. The anti-IL-15 Ab group exhibited a relatively delayed improvement in DA and LA waves by week 4. In contrast, the control IgG treatment did not lead to any improvement in the DA or LA responses (Figs. 3A, 4A). At the end of the treatment (week 2), the anti-IL-7 Ab group showed significantly better improvement from baseline in the amplitudes of DA a-waves and LA b-waves compared to both control IgG and the anti-IL-15 Ab treatments; however, 2 weeks after the treatment stopped (i.e., week 4), such differences disappeared (Figs. 3B, 4B). On the other hand, the anti-IL-15 Ab group showed a slower but progressive improvement, with a significantly increased amplitude of DA b-waves from baseline compared to both control IgG and the anti-IL-7 Ab groups at week 4 (Figs. 3B, 4B).

### Neutralization of IL-7 or IL-15 Is Similarly Effective in Eliminating Pathogenic Memory CD4^+^ T Cells in Chronic Uveitis

**Figure 5.**
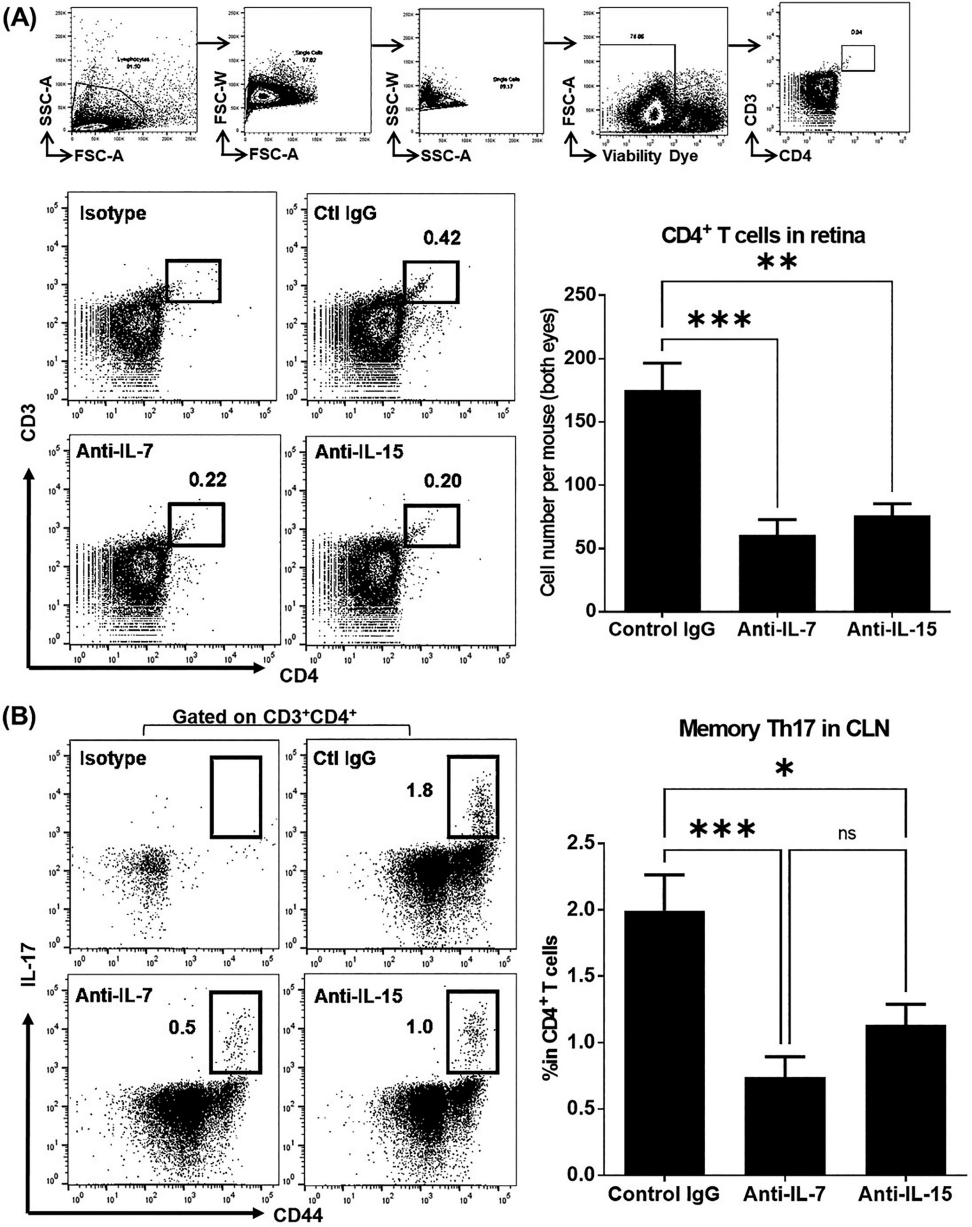
T-cell response in retina and draining lymph nodes in chronic uveitis. At week 4, retinal tissue and eye-draining CLN were collected and prepared for flow cytometric assessment. **(A)** The absolute number of CD4^+^ T cells infiltrated in the retinal tissue was extrapolated from the total number of retinal cells counted from both eyes of each mouse. The upper panel of plots shows the gating strategy for singlets and viability analysis. **(B)** The frequencies of CD44^hi^IL-17^+^ memory Th17 cells in CLN were evaluated. Representative flow cytometry plots are shown on the left, and bar summary graphs are shown on the right. Data shown are mean ± SEM of pooled three independent experiments. *n* = 10/group; *P < 0.05, **P < 0.01, ***P < 0.001.

We have previously demonstrated the pathogenic function of memory CD4^+^ T cells in chronic uveitis.^29^ We thus evaluated the T-cell response at week 4 after the treatment. Either anti-IL-7 Ab or anti-IL-15 Ab significantly reduced the retinal infiltration of CD4^+^ T cells, which exhibit unique memory phenotypes,^29^ compared to the control IgG treatment (Fig. 5A). Furthermore, the anti-IL-7 Ab and anti-IL-15 Ab treatment effectively decreased the memory Th17 cells (CD44^hi^IL-17^+^) in the eye-draining cervical lymph nodes (CLN) compared to the control IgG treatment (Fig. 5B).

## Discussion

Current treatments for chronic noninfectious uveitis are insufficient. More effective therapies that can disrupt the disease chronicity and prevent disease recurrence are desired. Grounded on our previous studies demonstrating the pathogenic roles of memory CD4^+^ T cells in chronic uveitis,^29^ as well as increased memory CD4^+^ T cells in the peripheral blood of uveitis patients^20^^,^^42^^,^^43^ and association of memory T-cell differentiation pathway with clinical disease from a large-scale bioinformatics analysis of uveitis patients data,^44^ the present study has further provided proof-of-concept evidence supporting a novel therapeutic strategy targeting the long-lived, pathogenic memory CD4^+^ T cells in chronic uveitis to achieve sustained disease remission. Overall, the two-week treatment with systemic anti-IL-7 Ab or anti-IL-15 Ab effectively reduced the memory CD4^+^ T cells in both the target tissue (retina) and its draining lymphoid tissues (serving as a reservoir for the retina-attacking “effector-memory” T cells that were capable of recirculating between lymphoid and non-lymphoid tissues), and thus ameliorated the disease severity assessed with fundoscopy and OCT examinations. In addition, the anti-IL-7 Ab treatment led to a rapid, and the anti-IL-15 Ab treatment led to a delayed improvement of retinal function assessed with ERG.

It has been long recognized that immunological memory is critical to the defense against pathogen reinfection, and it is the foundation for the protection conferred by vaccination, due to its long-term persistence, antigenic specificity, and ability to mount a rapid and enhanced response. Recently, increasing evidence has demonstrated that immunological memory also plays a critical role in autoimmune disorders and chronic inflammation.^45^^–^^48^ Therefore, the development of novel therapeutic approaches precisely targeting the pathogenic immune memory may lead to long-term remission or even cure of the disease. Among the major memory cell populations, CD4^+^ memory T cells are the major players in mediating chronic autoimmune inflammation, primarily through secreting IL-17. Studies on anti-IL-17 Ab in EAU have focused on the acute peak of the disease (two to three weeks after induction), which is dominated by short-lived effector Th17 cells that spontaneously decline as inflammation resolves.^20^^,^^49^ Neutralizing IL-17 secreted by effector Th17 during this acute period, along with the natural diminishment of the cellular source of IL-17, thus reduces disease severity. However, clinical trials in chronic uveitis showed that while anti-IL-17 treatment initially reduced disease severity, it failed to achieve sustained remission, the primary efficacy endpoint and the key goal in chronic uveitis treatment.^22^^,^^23^^,^^50^ The dominance of long-lived memory Th17 cells which persist without spontaneous diminishment for a long time in experimental chronic uveitis,^28^^,^^29^ supported by human data,^20^^,^^42^^,^^43^ can explain why IL-17 neutralization in humans offers only short-term relief: it does not eliminate these persisting memory cells that continuously produce IL-17.^30^ In our current study, we targeted these cells by depriving them of survival factors (IL-7 or IL-15),^30^ aiming to disrupt disease chronicity and achieve sustained disease amelioration. Given the high expression of IL-7R and IL-15R on the uveitogenic memory CD4^+^ T cells,^28^^,^^29^ we expected a similar effect of blocking either IL-7 or IL-15 in chronic uveitis. Our results showed that while anti-IL-7 led to significant disease improvement on both fundoscopic and OCT examinations, the anti-IL-15 only demonstrated a significant efficacy on OCT, along with a moderate efficacy on fundoscopic examination; however, blockade of IL-7 or IL-15 effectively suppressed the retinal infiltrating T cells in a similar degree. These findings suggest the inadequate dosages of the anti-IL-15 Ab used in the present study. A “step-dose” regimen for using IL-7 or IL-15 to expand T cells *in vivo* has been shown to efficiently act on respective receptors while minimizing potential toxicity,^51^^,^^52^ which may be borrowed in designing future Ab treatment studies. Previous studies on memory T cells (in “non-pathogenic” settings) indicate that IL-7 is more important than IL-15 in regulating the homeostasis of memory CD4^+^ T cells,^53^^–^^55^ and IL-15 is only essential for memory CD4^+^ T cell homeostasis when IL-7 is absent.^56^ In a subset of chronic uveitis patients (Vogt-Koyanagi-Harada disease), RNAseq analysis on the peripheral blood cells revealed that the gene for IL-7R is one of the highly expressed molecules in effector-memory CD4^+^ T cells.^43^ Taken together, it is conceivable that the relative contribution of IL-7 versus IL-15 in maintaining the pathogenic memory CD4^+^ T cells may depend on the history of antigen exposure, nature of the antigen, or anatomic location.

It is known that the IL-7 receptor is broadly expressed by multiple types of immune cells and lymphatic endothelial cells, and IL-7 plays crucial roles beyond in T cells, including in the development of B cells and innate lymphoid cells (ILC) and in the formation of tertiary lymphoid structures.^57^ It is thus possible that the in vivo suppression of memory T cells by systemic treatment with anti-IL-7 Ab may not be entirely due to direct suppression of memory T cells. As shown in Supplemental Figure S1, the treatment did not significantly affect the non-CD3^+^ cells (i.e., non-T cells, including B cells in the majority and a few other types of cells such as innate lymphoid cells and myeloid cells) in the secondary lymphoid organs, suggesting a low likelihood of major contribution of indirect mechanism to suppressed memory T cells by anti-IL-7 Ab treatment. It is currently acknowledged that the maintenance of memory T cells primarily requires cytokines such as IL-7 and IL-15 with little dependence on other cells,^30^^,^^58^ however, further in vitro experiments and in vivo local treatment with the anti-IL-7 Ab at the inflammatory site (such as intravitreal injection) may help to clarify the precise treatment targets in chronic uveitis.

Chronic uveitis primarily shows retinal infiltrates and structural damages, without significant optic disc edema, retinal blood vessel cuffing, or vitreous infiltration as seen in acute uveitis (i.e., the EAU model) in fundoscopic and OCT assessment, therefore, in our study we have modified the uveitis grading system by focusing on retinal tissue changes to better differentiate the intergroup differences. Our data show consistency between fundoscopic and OCT scoring. On OCT examination, HRF have emerged as a notable clinical biomarker for intraocular inflammation and other disease activity in a variety of retinal conditions such as retinitis pigmentosa,^59^ diabetic retinopathy,^35^ and age-related macular degeneration.^34^ In cases of chronic uveitis, depending on their structural features and location within the retina, HRF are thought to represent intraretinal exudates, infiltrating lymphocytes, or clustering of damaged photoreceptors/ RPE cells.^60^^–^^62^ As seen in our CAU model, the large clumps of foci mainly occupied the outer retina, which is consistent with the human findings,^63^ suggesting a pathogenic process initiated by the inflamed choroid plexus via the damaged barrier of the RPE layer. Large foci were also observed in the inner retina in some of the CAU mice, which has been used as an indicator for more severe disease.^33^ HRF have been considered in assessing the treatment response in patients with diabetic macular edema,^64^^,^^65^ and they have been observed to decrease with treatment in patients with non-infectious uveitic macular edema.^63^ Similarly, in the present study, the anti-IL-7 Ab and anti-IL-15 Ab treatment has been shown to decrease the number and size of the HRF effectively, further highlighting HRF as a biomarker for treatment response in chronic uveitis. One potential limitation in OCT scoring is that HRF in deeper tissues might be masked by overlying inflammation (back shadowing), which may lead to underestimation or misinterpretation of the severity and extent of retinal changes. More advanced imaging techniques or image enhancement tools may be required to improve the reliability of OCT scoring.

The visual functional assessment on the retina showed that the anti-IL-7 Ab effectively improved DA a-waves and LA b-waves, but not DA b-waves on the full-field ERG recordings, indicating the relative resistance to recovery for the impaired bipolar cells that relay rod signals.^66^ In addition, the treatment effect took place between one to two weeks after the initiation of the treatment, indicating that the restoration of retinal function occurs earlier than the structural improvement in response to treatment. On the other hand, during the disease development, retinal dysfunction also took precedence over structural damage.^29^ These findings suggest that retinal function is more sensitive to changes in the microenvironment. However, such early improvement in retinal function with the anti-IL-7 Ab treatment was not sustained, which contrasted with the more lasting treatment effects on retinal structures, suggesting that evaluation of retinal structures alone may not be sufficient to accurately predict the treatment response and thus ERG assessment is essential in preclinical studies assessing novel therapeutic strategies for uveitis. In our case, we may need to prolong the treatment period, as conducted in human trials (usually months to years of treatment), to achieve a more lasting effect on the recovery of retinal function. In the case of anti-IL-15 Ab treatment, our results showed a relative shower effect on ERG as evidenced by significantly improved DA b-waves observed at four weeks after the initiation of the treatment, suggesting the necessity of increasing the dosage of the anti-IL-15 Ab for achieving optimal efficacy.

Various strategies of blocking IL-7-IL7R signaling have been previously tested in autoimmune or chronic inflammatory models, either at the disease induction or sustention stage. Genetic suppression of IL-7 expression in mice led to reduced development of experimental autoimmune encephalomyelitis, a murine model for human multiple sclerosis.^67^ In an adoptive transfer model of mouse colitis mimicking human inflammatory bowel disease, IL-7 knockout mice did not develop chronic colitis.^68^ Systemic anti-IL-7 Ab treatment effectively suppressed the delayed allograft rejection and allogenic memory T cells in mouse models of heart or skin transplantation.^39^ In addition to IL-7, IL-7 receptor has also been targeted. IL-7R-deficient mice showed resistance to the induction of experimental autoimmune encephalomyelitis and T cell response.^69^ Systemic anti-IL7R Ab treatment significantly suppressed the induction and progression of collagen-induced arthritis, a mouse model of human rheumatoid arthritis.^70^ Another study showed that toxin-conjugated anti-IL-7R Ab treatment completely ameliorated established, ongoing colitis in the mouse model.^71^

A major potential side effect of blocking IL-7 or IL-15 systemically could be the compromise of the homeostasis of naive T cells or non-pathogenic memory T cells (such as protective memory derived from past vaccination) which also express receptors for both cytokines.^31^^,^^55^ However, with the protocol we used, we did not find any significant reduction of total CD4^+^ or CD8^+^ T cell pool in animals treated with anti-IL-7 Ab or anti-IL-15 Ab compared to the control IgG treatment (Supplementary Fig. S1). Further subsets analysis showed a nonsignificant decrease of memory T-cell fraction among total CD4^+^ or CD8^+^ T cell pool of CLN in both treatment groups with anti-IL-15 Ab imposing a larger effect (Supplementary Fig. S2); such moderate decrease could attribute to diminished pathogenic memory CD4^+^ T cells and mild effect on non-pathogenic memory T cells. However, neither Ab treatment led to a reduction of memory T-cell fraction in the spleen (Supplementary Fig. S3), suggesting that our treatment regimen did not elicit a significant global effect on all memory T cells. Instead, the anti-IL-7 Ab treatment group showed an increased trend in memory CD4^+^ T cell fraction in spleen, which could be due to the relative reduction of naive CD4^+^ T cell fraction (Supplementary Fig. S3) that is potentially impacted by the Ab treatment. The safety of targeting IL-7 or IL-7R has been studied in human patients. Clinical trials in type 1 diabetes showed that appropriate dosages of systemic anti-IL-7R Ab treatment selectively inhibited memory T cells while preserving naive T cells or regulatory T cells (Treg), as well as protective memory from past vaccinations.^72^^,^^73^ In addition, another safety trial of systemic anti-IL-7R Ab in healthy subjects showed that treatment did not lead to any discernible impact on peripheral T cell subsets.^74^ Currently, a new drug targeting IL-7 is under development for the treatment of multiple sclerosis (ClinicalTrials.gov NCT05131971).

Our study represents the first to evaluate the novel therapeutic approach of specifically targeting pathogenic memory CD4^+^ T cells in chronic uveitis and has laid the ground for further preclinical assessment of blocking IL-7 or IL-15. Future investigation on optimizing dosage and treatment duration with longer follow-up using the CAU model will facilitate the translation of this promising strategy for chronic noninfectious uveitis.

## Supplementary Material

Supplement 1
