# Supplementary material for: Comparative Efficacy of Interleukin-7 and -15 Blockade in Alleviating Experimental Chronic Uveitis and Suppressing Pathogenic Memory CD4+ T Cells
Source: Invest Ophthalmol Vis Sci. 2025 May 5;66(5):9. doi: 10.1167/iovs.66.5.9 (PMC12060075; doi:10.1167/iovs.66.5.9)
Supplement: Supplement 1 [file iovs-66-5-9_s001.pdf]

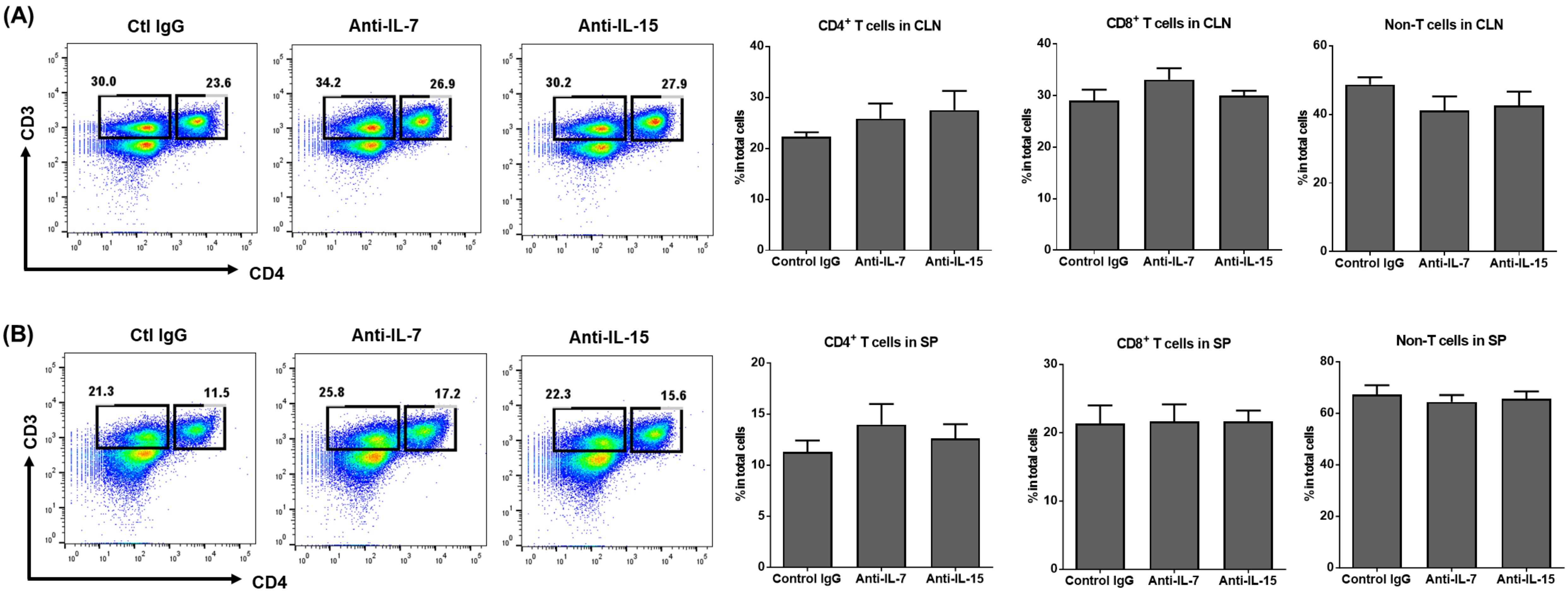

Figure S1. Impact of Ab treatment on total $CD4^+$ and $CD8^+$ T cell pools and non-T cell pools in secondary lymphoid organs (SLO)
At week 4, peripheral eye-draining cervical lymph nodes (CLN) and spleen (SP) were collected and prepared for flow cytometric assessment. Frequencies of total $CD4^+CD3^+$ and $CD4^-CD3^+$ ($CD8^+$) T cells, as well as $CD3^-$ non-T cells, in the ELN **(A)** and SP **(B)** were evaluated. Representative flow cytometry plots are shown on the left, and bar graphs summarizing the frequencies are shown on the right. Data shown are mean ± SEM of pooled three independent experiments. n = 5-6/group.

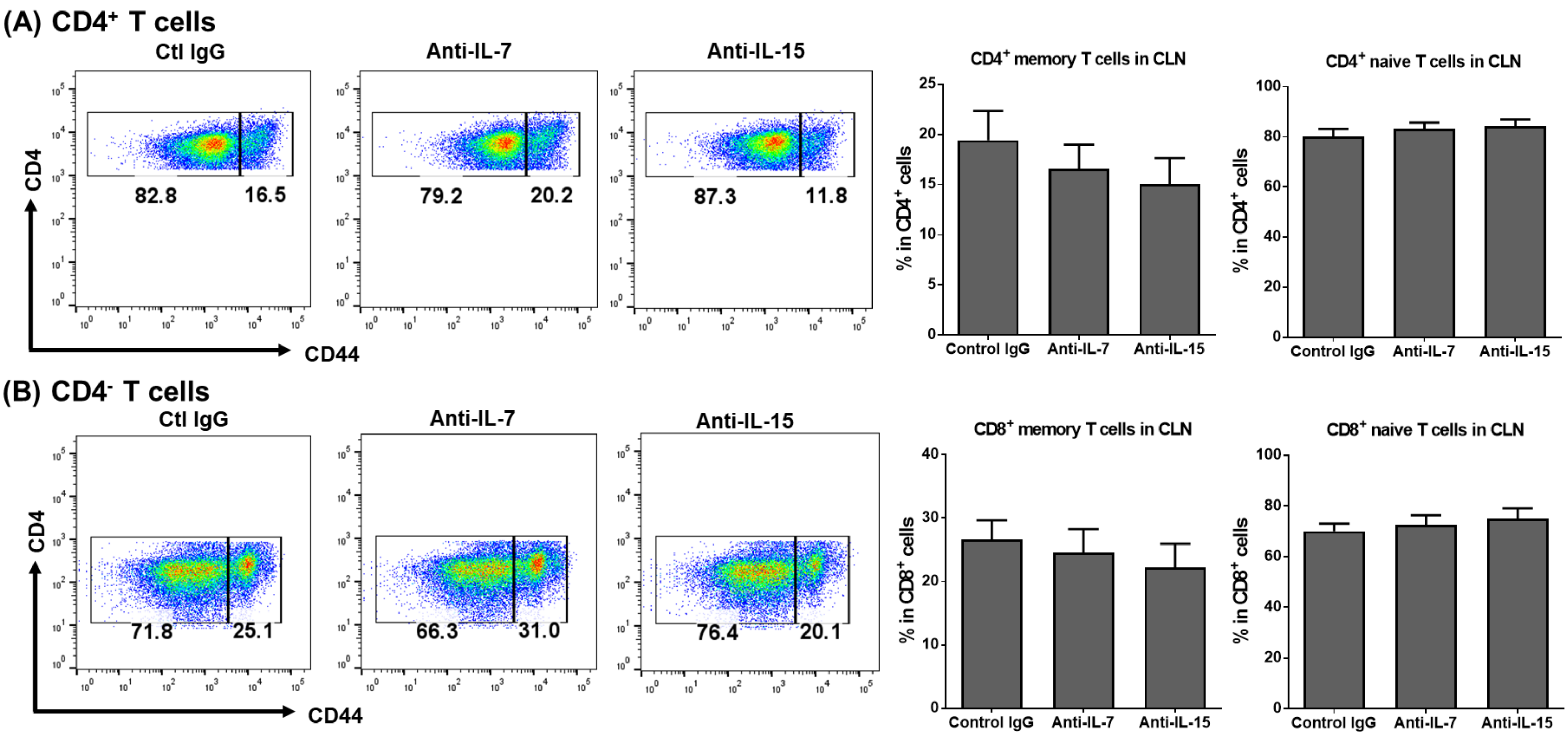

Figure S2. Impact of Ab treatment on CD4⁺ and CD8⁺ memory and naive T subsets in eye-draining cervical lymph nodes (CLN)
T memory ($CD44^+$) and naive ($CD44^-$) subsets were gated from total $CD4^+$ **(A)** or $CD8^+$ ($CD4^-$) **(B)** T cells in Fig. S1. Representative flow cytometry plots are shown on the left, and bar graphs summarizing the frequencies are shown on the right. Data shown are mean ± SEM of pooled three independent experiments. n = 5-6/group.

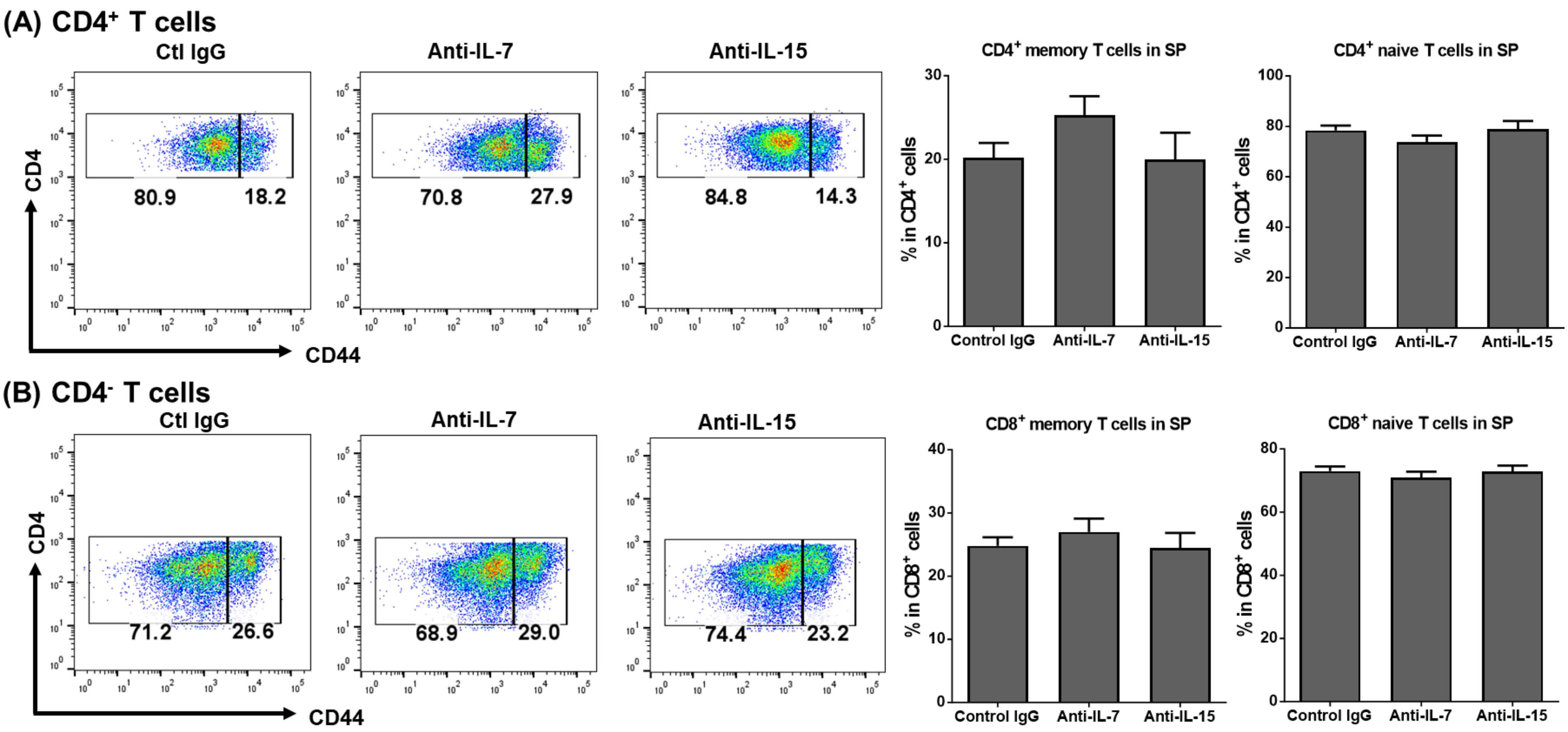

Figure S3. Impact of Ab treatment on CD4⁺ and CD8⁺ memory and naive T subsets in spleen (SP)

T memory (CD44⁺) and naive (CD44⁻) subsets were gated from total CD4⁺ **(A)** or CD8⁺ (CD4⁻) **(B)** T cells in Fig. S1. Representative flow cytometry plots are shown on the left, and bar graphs summarizing the frequencies are shown on the right. Data shown are mean $\pm$ SEM of pooled three independent experiments. n = 5-6/group.
